# siRNA silencing of PD-1 ligands on dendritic cell vaccines boosts the expansion of minor histocompatibility antigen-specific CD8^+^ T cells in NOD/SCID/IL2Rg(null) mice

**DOI:** 10.1007/s00262-015-1668-6

**Published:** 2015-02-28

**Authors:** Anniek B. van der Waart, Hanny Fredrix, Robbert van der Voort, Nicolaas Schaap, Willemijn Hobo, Harry Dolstra

**Affiliations:** 1 Department of Laboratory Medicine, Laboratory of Hematology, Radboud University Medical Center, Geert Grooteplein 8, P.O. Box 9101, 6500 HB Nijmegen, The Netherlands; 2 Department of Hematology, Radboud University Medical Center, Nijmegen, The Netherlands

**Keywords:** Adoptive cell therapy, Dendritic cell, GVT, Leukemia, Minor histocompatibility antigen, PD-L

## Abstract

Allogeneic stem cell transplantation (allo-SCT) can be a curative therapy for patients suffering from hematological malignancies. The therapeutic efficacy is based on donor-derived CD8^+^ T cells that recognize minor histocompatibility antigens (MiHAs) expressed by patient’s tumor cells. However, these responses are not always sufficient, and persistence and recurrence of the malignant disease are often observed. Therefore, application of additive therapy targeting hematopoietic-restricted MiHAs is essential. Adoptive transfer of MiHA-specific CD8^+^ T cells in combination with dendritic cell (DC) vaccination could be a promising strategy. Though effects of DC vaccination in anti-cancer therapy have been demonstrated, improvement in DC vaccination therapy is needed, as clinical responses are limited. In this study, we investigated the potency of program death ligand (PD-L) 1 and 2 silenced DC vaccines for ex vivo priming and in vivo boosting of MiHA-specific CD8^+^ T cell responses. Co-culturing CD8^+^ T cells with MiHA-loaded DCs resulted in priming and expansion of functional MiHA-specific CD8^+^ T cells from the naive repertoire, which was augmented upon silencing of PD-L1 and PD-L2. Furthermore, DC vaccination supported and expanded adoptively transferred antigen-specific CD8^+^ T cells in vivo. Importantly, the use of PD-L silenced DCs improved boosting and further expansion of ex vivo primed MiHA-specific CD8^+^ T cells in immunodeficient mice. In conclusion, adoptive transfer of ex vivo primed MiHA-specific CD8^+^ T cells in combination with PD-L silenced DC vaccination, targeting MiHAs restricted to the hematopoietic system, is an interesting approach to boost GVT immunity in allo-SCT patients and thereby prevent relapse.

**Electronic supplementary material:**

The online version of this article (doi:10.1007/s00262-015-1668-6) contains supplementary material, which is available to authorized users.

## Introduction

Allogeneic stem cell transplantation (allo-SCT) can be a curative therapy for patients suffering from high-risk hematological malignancies [1–3]. Donor-derived CD8^+^ T cells play an important role in the therapeutic effect of this treatment, the so-called graft-versus-tumor (GVT) reaction. These CD8^+^ T cells can be directed against minor histocompatibility antigens (MiHAs) that are human leukocyte antigen (HLA)-bound peptides derived from polymorphic genes that differ between patient and donor [4, 5]. Importantly, a selective GVT response occurs when the expression of these MiHAs is restricted to hematopoietic tissues [6]. Previously, we showed that patients transplanted with a stem cell graft from a MiHA-mismatched donor (patient expressing the MiHA, whereas the donor does not) have an improved relapse-free survival (RFS) [7]. This RFS was especially increased in patients in whom MiHA-specific CD8^+^ T cells could be detected. However, these responses are infrequent, and persistence or recurrence of the malignant disease is often observed. This emphasizes the need to further boost GVT-selective MiHA-specific CD8^+^ T cell responses in allo-SCT patients.

One of the current additive treatment options is donor lymphocyte infusion (DLI). However, this non-selected donor T cell product contains only a limited number of naive T cells recognizing hematopoietic-restricted MiHAs that are able to contribute a specific GVT effect. Moreover, T cells present in the DLI can also recognize polymorphic antigens expressed by healthy host tissues, thereby causing graft-versus-host disease (GVHD) [8, 9], the major cause of morbidity and mortality post-allo-SCT. Therefore, adoptive T cell therapy with purified and expanded donor CD8^+^ T cells selective for MiHAs restricted to the hematopoietic system could lead to a more efficacious GVT effect without promoting GVHD.

T cell responses can be initiated, supported and boosted by dendritic cells (DCs). These professional antigen-presenting cells can activate T cells upon presentation of a peptide in concordance with co-stimulatory signals, which is dependent on the balance between co-inhibitory and co-stimulatory interactions. Two of the co-inhibitory ligands involved in this process are program death ligand (PD-L) 1 and PD-L2. We have shown previously that their receptor, program death (PD)-1, is highly expressed on MiHA-specific CD8^+^ T cells after allo-SCT, possibly due to chronic antigen exposure as observed in chronic viral infections [10]. This high PD-1 expression was involved in the functional inhibition of MiHA-specific CD8^+^ T cells. Importantly, we demonstrated that interference with this pathway resulted in enhanced expansion of MiHA-specific CD8^+^ T cells of allo-SCT patients in vitro [10–12]. Either by using blocking antibodies against PD-1 or PD-L1 [10] or by silencing the inhibitory ligands PD-L1 and PD-L2 on DCs, which results in more stimulatory DCs, we augmented the expansion of MiHA-specific CD8^+^ T cells of allo-SCT patients in vitro. As PD-1/PD-L1 blocking antibodies early after allo-SCT might increase the risk of GVHD [13], adoptive transfer of tumor-reactive T cells in combination with DC vaccination would be a safer adjuvant therapeutic approach early post-allo-SCT. These DCs can maintain and expand the adoptively transferred MiHA-specific CD8^+^ T cells, which will contribute to the reduction in the tumor load and generation of an immunological memory to control relapse. Though some clinical responses of DC vaccination in anti-cancer therapy have been demonstrated, improvement in DC vaccination therapy is needed [14–19]. By augmenting the stimulatory capacity of DCs, via silencing of the co-inhibitory ligands PD-L1 and PD-L2, we aim to superiorly boost and augment adoptively transferred MiHA-specific CD8^+^ T cell responses in vivo and thereby improve survival. In this study, we show that using monocyte-derived DCs, functional MiHA-specific CD8^+^ T cells can be primed and expanded in vitro, which is augmented by silencing the co-inhibitory ligands PD-L1 and PD-L2 on the DCs. Moreover, we demonstrate in immunodeficient mice that DC vaccination can boost the expansion of adoptively transferred MiHA-specific CD8^+^ T cells, in which PD-L silenced DCs show their superior stimulatory potential.

## Materials and methods

### Donor material and MiHA genotyping

Peripheral blood mononuclear cells (PBMCs) were isolated from HLA-A2 and HLA-B7 positive buffy coats (Sanquin Blood Supply Foundation, Nijmegen, the Netherlands) after written informed consent. PBMCs were genotyped for the MiHAs HA1 [20], HA2 [21], LRH1 [22] and ARHGDIB [23] with the KASPar assay system (KBioscience, Hoddesdon, UK), a fluorescence-based competitive allele-specific PCR using non-labeled primers. Details of this method are available at http://www.kbioscience.co.uk.

### siRNAs targeting PD-L1 and PD-L2

PD-L1 and PD-L2 siRNAs were kindly provided by dr. Anna Borodovsky from Alnylam Pharmaceuticals and produced as described previously [12, 24]. The duplex sequences were as follows: PD-L1, 5′AGAccuuGAuAcuuucAAAdTsdT-3′ (sense), 5′-UUUGAAAGuAUcAAGGUCUdTsdT-3′ (anti-sense); PD-L2, 5′-AuAAcAGccAGuuuGcAAAdTsdT-3′ (sense), 5′-UUUGcAAACUGGCUGUuAUdTsdT-3′ (anti-sense). As a negative control, a siRNA duplex for luciferase was used, with the sequence 5′-cuuAcGcuGAGuAcuucGAdTsdT-3′ (sense) and 5′-UCGAAGuACUcAGCUcAGCGuAAGdTsdT-3′ (anti-sense). Small case represents 2′-*O*-methyl-modified residues.

### DC culture and PD-L silencing

Monocytes were isolated from PBMCs via plastic adherence in tissue culture flasks (Greiner Bio-One, Alphen a/d Rijn, the Netherlands). Intermediate DCs were generated by culturing monocytes in X-VIVO15 medium (Lonza, Verviers, Belgium) supplemented with 2 % human serum (HS; PAA laboratories, Pasching, Austria), 500 U/ml interleukin (IL)4 and 800 U/ml granulocyte macrophage colony-stimulating factor (GM-CSF, both Immunotools, Friesoythe, Germany). After 3 days, intermediate DCs were supplemented with fresh medium and cytokines or were transferred to 6-well plates and further cultured at 0.5 × 10^6^/ml X-VIVO15 supplemented with 2 % HS, 500 U/ml IL4 and 800 U/ml GM-CSF. At day 7, maturation was induced in X-VIVO15/2 % HS containing 5 ng/ml IL1ß, 15 ng/ml IL6, 20 ng/ml tumor necrosis factor (TNF)α (all Immunotools) and 1 µg/ml prostaglandin E2 (PGE2) (Pharmacia and Upjohn, Bridgewater, NJ, USA). At day 9, mature DCs were harvested, phenotyped and used in experiments. PD-L1 and PD-L2 were silenced using siRNAs and delivered via standard methods as described previously [11, 12] (Roeven et al., Journal of Immunotherapy, accepted for publication). Previously, we showed that PD-L1/PD-L2 siRNA silencing did not affect expression of the cell surface markers CD14, CD80, CD83, CD86, CCR7, MHC-I and MHC-II [12].

### Priming of MiHA-specific CD8^+^ T cells

Peripheral blood lymphocytes (PBLs), CD8^+^ T cells, naive or effector memory CD8^+^ T (Tn and Tem, respectively) cells were used in MiHA-specific CD8^+^ T cell priming experiments as indicated. CD8^+^ T cells were isolated using MACS microbeads (Miltenyi Biotec, Bergisch Gladbach, Germany) according to the manufacturer’s guidelines. To isolate Tn or Tem cells, CD3^+^CD8^+^CD45RA^+^CCR7^+^ and CD3^+^CD8^+^CD45RA^−^CCR7^−^ cells were sorted after CD8 MACS isolation using the FACS Altra (Beckman Coulter, Fullerton, CA, USA). Priming was performed by co-culturing PBLs, CD8^+^, Tn or Tem cells with mature PD-L silenced or control siRNA-treated DCs, loaded with 5 µM MiHA peptide. Cells were cultured for 7 days in IMDM/10 % HS at a T cell:DC ratio of 1:0.1. At day 2 and 5, IL2 (50 U/ml, Chiron CA, USA) and IL15 (5 ng/ml, Immunotools) were added. At day 7, T cells were analyzed by flow cytometry and when indicated re-stimulated for an additional week.

### Flow cytometry

Phenotype and maturation state of DCs were analyzed by staining with the following antibodies against PD-L1 (clone MIH1), PD-L2 (clone MIH18, both from Becton–Dickinson, Franklin Lakes, NJ, USA) and isotype control IgG1 (Dako). Cytomegalovirus (CMV), LRH1, HA1, HA2 and ARHGDIB-specific CD8^+^ T cells were detected by staining cells with tetramers containing the corresponding peptide (CMV.B7: RPHERNGFTVL; LRH1.B7: TPNQRQNVC; HA1.A2: VLHDDLLEA; HA2.A2: YIGEVLVSV; ARHGDIB.B7: LPRACWREA). Tetramers were kindly provided by prof. Dr. Frederik Falkenburg (Department of Hematology, Leiden University Medical Center, Leiden, the Netherlands). Cells were incubated with 0.15–0.2 μg APC and PE labeled tetramer for 15 min at room temperature. Subsequently, cells were labeled with antibodies against the surface markers: mouse CD45 (clone 30-F11, Biolegend, San Diego, CA, USA), human CD45 (clone J33, Beckman Coulter), CD8 (clone 3B5, Life Technologies, Grand Island, NY, USA), CD3 (clone UCHT1, Biolegend), CD45RA (clone 2H4LDH11LDB9, Beckman Coulter), CCR7 (clone 150503, R&D Systems) and CD28 (Biolegend) for 30 min at 4 °C. After washing with PBS/0.5 % bovine serum albumin (BSA; Sigma, St Louis, MO, USA), cells were resuspended in washing buffer containing 1:5000 Sytox Blue (Life Technologies) to discriminate dead cells and analyzed using the Cyan-ADP 9 color analyzer or the Gallios (both Beckman Coulter). Analysis was performed using Summit (Dako) and Kaluza software (Beckman Coulter).

### MiHA-specific CD8^+^ T cell functionality assay

Primed MiHA-specific CD8^+^ T cells were tested for their IFNγ production upon peptide rechallenge. Therefore, cells were restimulated overnight with MiHA peptide (5 µM) in the presence of brefeldin A (1 µg/ml, BD Biosciences). The following day, extracellular staining by MiHA tetramers and antibodies recognizing CD3 and CD8 was performed, followed by intracellular staining for IFNγ. For this, cells were resuspended in 4 % paraformaldehyde (PFA) and incubated for 10 min at RT. Then, cells were incubated in 0.1 % saponin (Sigma) buffer containing 10 % fetal calf serum (Integro, the Netherlands) for 10 min at RT. This was followed by intracellular staining for anti-CD137 (clone 41BB, Biolegend) and anti-IFNγ (clone B27, BD Biosciences) for 30 min at 4 °C after which cells were fixed in 1 % PFA and measured on the Gallios flow cytometer.

### Antigen-specific CD8^+^ T cell expansion in vivo by DC vaccination

NOD/SCID/IL2Rγ^null^ (NSG) mice were originally purchased from Jackson Laboratories, and housed and bred in the Radboud university medical center (Radboudumc) Central Animal Laboratory. Female NSG mice were used from 6 to 20 weeks of age. All animal experiments were approved by the Animal Experimental Committee of the Radboudumc and were conducted in accordance with institutional and national guidelines, permit number 10300.

Mice were i.v. injected with 10–15 × 10^6^ (cultured) PBLs containing antigen-specific CD8^+^ T cells and weekly vaccinated with 0.25–0.5 × 10^6^ peptide-loaded autologous control or PD-L silenced DCs (i.p.). To support antigen-specific CD8^+^ T cells, 0.5 μg recombinant human IL15 (Miltenyi, i.e., 2500 units) was administered i.p. every 2–3 days when indicated. At indicated time points, mice were killed and organs were analyzed for presence of antigen-specific CD8^+^ T cells by flow cytometry.

### Statistical analysis

The data were analyzed using Graphpad Prism 4. Statistical significance was analyzed using a Student’s *t* test or one-way analysis of variance (ANOVA) followed by a Bonferroni post hoc test as indicated in the figure legends. *p* < 0.05 was considered to be significant.

## Results

### Highly efficient priming and expansion of MiHA-specific CD8^+^ T cells by PD-L silenced DCs

**Fig. 1:**
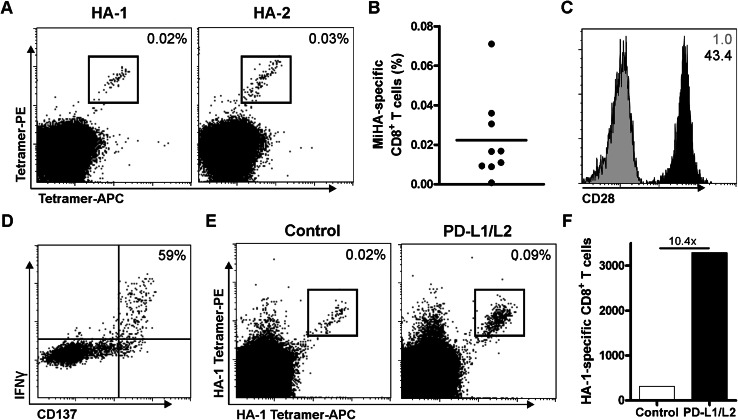
Priming of functional MiHA-specific CD8^+^ T cells is enhanced by PD-L silenced DCs. CD8^+^ T cells were co-cultured with MiHA peptide-loaded allogeneic DCs and analyzed at day 7 by flow cytometry for the presence of MiHA-specific CD8^+^ T cells. **a** Representative FACS plot of two independent cultures. The *numbers* in the FACS plots represent the percentage of MiHA-specific CD8^+^ T cells within the total CD3^+^CD8^+^ T cell population. **b** Combined data of nine independent cultures. **c** Representative FACS plot of CD28 expression (*black*) on MiHA-specific CD8^+^ T cells at day 7 of culture, isotype control in *gray*. The *numbers* in the FACS plots represent mean fluorescence intensity (MFI). One out of four independent cultures is shown. **d** Representative FACS plot of cultured cells at day 7, which were overnight re-stimulated with peptide followed by intracellular staining for IFNγ and CD137. The *number* in the FACS plots represents the percentage of IFNγ^+^ cells within CD137^hi^ CD8^+^ T cells. **e**–**f** CD8^+^ T cells were cultured for two consecutive weeks with HA-1 peptide-loaded PD-L silenced or control DCs (relative expression: PD-L1 6 %, PD-L2 23 %), and analyzed at day 14 by flow cytometry for the presence of MiHA-specific CD8^+^ T cells. FACS plot (**e**) and absolute cell numbers (**f**) of cultures with control or PD-L silenced DCs, *n* = 1

Previously, we showed that PD-L silenced DCs augment expansion of MiHA-specific CD8^+^ effector memory cells from transplanted patients [11, 12]. To study the potency of PD-L silenced DCs in the expansion of MiHA-specific CD8^+^ T cells from the naive repertoire, in vitro priming experiments were performed using CD8^+^ T cells from MiHA-negative donors. These T cells were co-cultured with MiHA peptide-loaded HLA-A2^+^ or B7^+^ DCs, and weekly analyzed by flow cytometry. At day 7, we observed an average of 0.02 % MiHA-specific CD8^+^ T cells in the cultures (Fig. 1a–b; Table 1). Phenotypical analysis of these T cells showed high expression of the co-stimulatory molecule CD28 (Fig. 1c). In addition, the functionality of these T cells was determined by overnight peptide rechallenge followed by intracellular staining for IFNγ. Upon this antigen-specific stimulation, we observed production of IFNγ, but only in cells expressing the activation marker CD137 (Fig. 1d). Within this activated T cell population, 59 % of the cells produced IFNγ, indicating high functionality of the expanded MiHA-specific CD8^+^ T cells. Combined, this showed the feasibility of this method using DCs to ex vivo prime and generate highly functional MiHA-specific CD8^+^ T cells.

**Table 1:** Primed MiHA-specific CD8^+^ T cells

Donor	MiHA	MiHA-specific CD8^+^ T cells (%)^a^
1	HLA-A2.HA1	0.009
2	HLA-A2.HA1	0.009
3	HLA-A2.HA1	0.011
4	HLA-A2.HA1	0.017
4	HLA-A2.HA2	0.031
5	HLA-B7.LRH1	0.017
6	HLA-B7.LRH1	0.001
6	HLA-B7.ARHGDIB	0.036
7	HLA-B7.ARHGDIB	0.071

^a^Percentage of total CD8^+^ T cells

Next, we investigated whether PD-L silenced DCs would be more effective in priming MiHA-specific CD8^+^ T cells. Therefore, we co-cultured CD8^+^ T cells with peptide-loaded control or PD-L silenced DCs. Though MiHA-specific CD8^+^ T cells could be expanded by control DCs, the use of PD-L silenced DCs strongly augmented the expansion of MiHA-specific CD8^+^ T cells within 2 weeks of culture (Fig. 1e). This resulted in a tenfold increase in the absolute number of MiHA-specific CD8^+^ T cells (Fig. 1f). These data show the superior potency of PD-L silenced DCs in the priming and expansion of MiHA-specific CD8^+^ T cells from MiHA-negative donors.

**Fig. 2:**
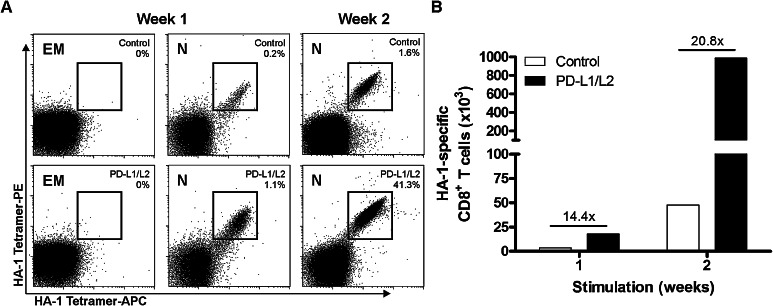
MiHA-specific CD8^+^ T cell priming and expansion from the naive repertoire is augmented by PD-L silenced DCs. Naïve (N, CD45RA^+^CCR7^+^) and effector memory (EM, CD45RA^−^CCR7^−^) CD8^+^ T cells were sorted and cultured for two consecutive weeks with HA-1 peptide-loaded PD-L silenced or control DCs (relative expression: PD-L1 7 %, PD-L2 12 %). The percentage (**a**) and cumulative numbers (**b**) of HA-1-specific CD8^+^ T cells obtained after stimulation with PD-L silenced or control DCs

To exclude that this observation could be attributed to the expansion of antigen-experienced MiHA-specific CD8^+^ effector memory T cells developed during pregnancy [25], both naive and effector memory CD8^+^ T cells were sorted from a MiHA^−^ donor, after which they were co-cultured with MiHA peptide-loaded control or PD-L silenced DCs. Again, highly efficient MiHA-specific CD8^+^ T cell priming was observed, but only in co-cultures that started with naive T cells (Fig. 2a). In co-cultures containing effector memory T cells, no MiHA-specific CD8^+^ T cells could be expanded. Although MiHA-specific CD8^+^ T cells primed from the naive repertoire by control DCs could be expanded to 1.6 % in 2 weeks, the use of PD-L silenced DCs resulted in augmented numbers of MiHA-specific CD8^+^ T cells (Fig. 2a). Notably, these cells expanded to more than 40 % of MiHA-specific CD8^+^ T cells after 1 week of re-stimulation. Together, this resulted up to 20-fold increase in MiHA-specific CD8^+^ T cells after 2 weeks of culture (Fig. 2b). These data show the superior potential of PD-L silenced DCs in the priming and expansion of MiHA-specific CD8^+^ T cell responses from the naive repertoire.

### Expansion of antigen-specific T cells by DC vaccination in NSG mice

**Fig. 3:**
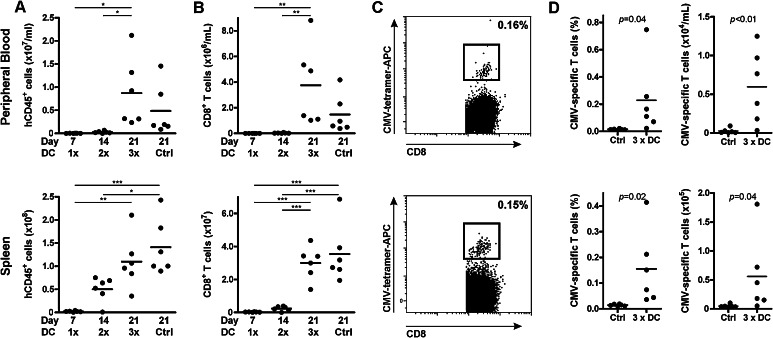
CMV-specific CD8^+^ T cell expansion by DC vaccination in vivo. Peripheral blood lymphocytes containing 2000 CMV-specific CD8^+^ T cells were injected in NSG mice and stimulated with no (ctrl), one, two or three peptide-loaded (5 µM) DC vaccinations at weekly intervals. Mice were killed 7 days after their last DC vaccination or at the end of the experiment (ctrl). Peripheral blood and spleen were analyzed by flow cytometry for human CMV-specific CD8^+^ T cells. Engraftment of human cells (**a**) and CD8^+^ T cell (**b**). Statistical analysis was performed using one-way ANOVA followed by a Bonferroni post hoc test. **c** Representative FACS plot of CMV-specific CD8^+^ T cells after three DC vaccinations. The *numbers* in the FACS plots represent the percentage of CMV-specific CD8^+^ T cells within the total CD3^+^CD8^+^ T cell population. **d** Percentage and absolute cell numbers of CMV-specific CD8^+^ T cells of mice non-vaccinated, or vaccinated three times. Statistical analysis was performed using a one-tailed Student’s *t* test. *p < 0.05; **p < 0.01; ***p < 0.001

To investigate the stimulatory capacity of PD-L silenced DCs on supporting and boosting adoptively transferred T cells in vivo, we generated a mouse model in which NSG mice were injected with PBLs containing antigen-experienced CD8^+^ T cells. Subsequently, mice were vaccinated weekly with peptide-loaded DCs. These mice received no, one, two or three DC vaccinations at weekly intervals, and mice were killed 1 week after their last vaccination. Mice receiving no DC vaccination were killed at the end of the experiment, similar to the group receiving three DC vaccines. Engraftment of the adoptively transferred human cells increased in time from 0.02 ± 0.09 × 10^6^/ml at week 1 to 8.7 ± 7.4 × 10^6^ human cells/ml at week 3 in peripheral blood (PB, mean ± SD, Fig. 3a). In addition, in spleen we observed an increase from 2.2 ± 1.3 to 110 ± 58 × 10^6^ human cells. This human engraftment reflected the increase in CD8^+^ T cell numbers in both PB and spleen (Fig. 3b). Notably, the DC vaccinations did not affect engraftment levels or consistency, as similar numbers were observed between mice receiving no or three DC vaccinations. Though in some mice receiving only one DC vaccination low numbers of CMV-specific CD8^+^ T cells could be detected, levels were generally below the detection limit (data not shown). With increasing engraftment of human cells in time, boosted CMV-specific CD8^+^ T cells could be clearly detected in mice receiving three DC vaccinations. In these mice, a CMV-specific CD8^+^ T cell population was detected up to 0.75 % of the total CD8^+^ T cell compartment in both spleen and PB. The absolute CMV-specific CD8^+^ T cells number in DC vaccinated mice was 56 ± 66 × 10^3^ in spleen and 6.0 ± 4.7 × 10^3^/ml in PB (Mean ± SD, Fig. 3c–d), which already in these two organs was 31 times higher than the infused number of CMV-specific CD8^+^ T cells, showing robust expansion of antigen-specific CD8^+^ T cells in vivo. In mice receiving no DC vaccination, CMV-specific CD8^+^ T cells were undetectable at time of analysis (day 21). These data demonstrate that this mouse model can be used to evaluate DC-mediated expansion of adoptively transferred antigen-specific CD8^+^ T cells in a humanized setting.

### PD-L silenced DCs enhanced expansion of adoptively transferred MiHA-specific CD8^+^ T cell

To study the stimulatory potency of PD-L silenced DCs in vivo, we first confirmed the stability of the silencing under inflammatory conditions. For this, we co-cultured DCs in the presence of IFNγ. Additionally, as the mouse model includes IL15 injections to support the survival and expansion of antigen-specific T cells, the effect of this low-dose IL15 was also investigated. For this, PD-L silenced DCs were generated, expressing only 5.4 % of PD-L1 and 1.4 % of PD-L2 compared to their control DCs (Supplementary Figure 1A). After 48 h of culture in the presence of IFNγ and/or IL15, flow cytometry analysis revealed no effects on expression of PD-L1 or PD-L2 on either PD-L silenced or control DCs (Supplementary Figure 1B). Concluding, silencing of PD-L on DCs results in a co-stimulatory phenotype, which is stably retained under inflammatory conditions.

**Fig. 4:**
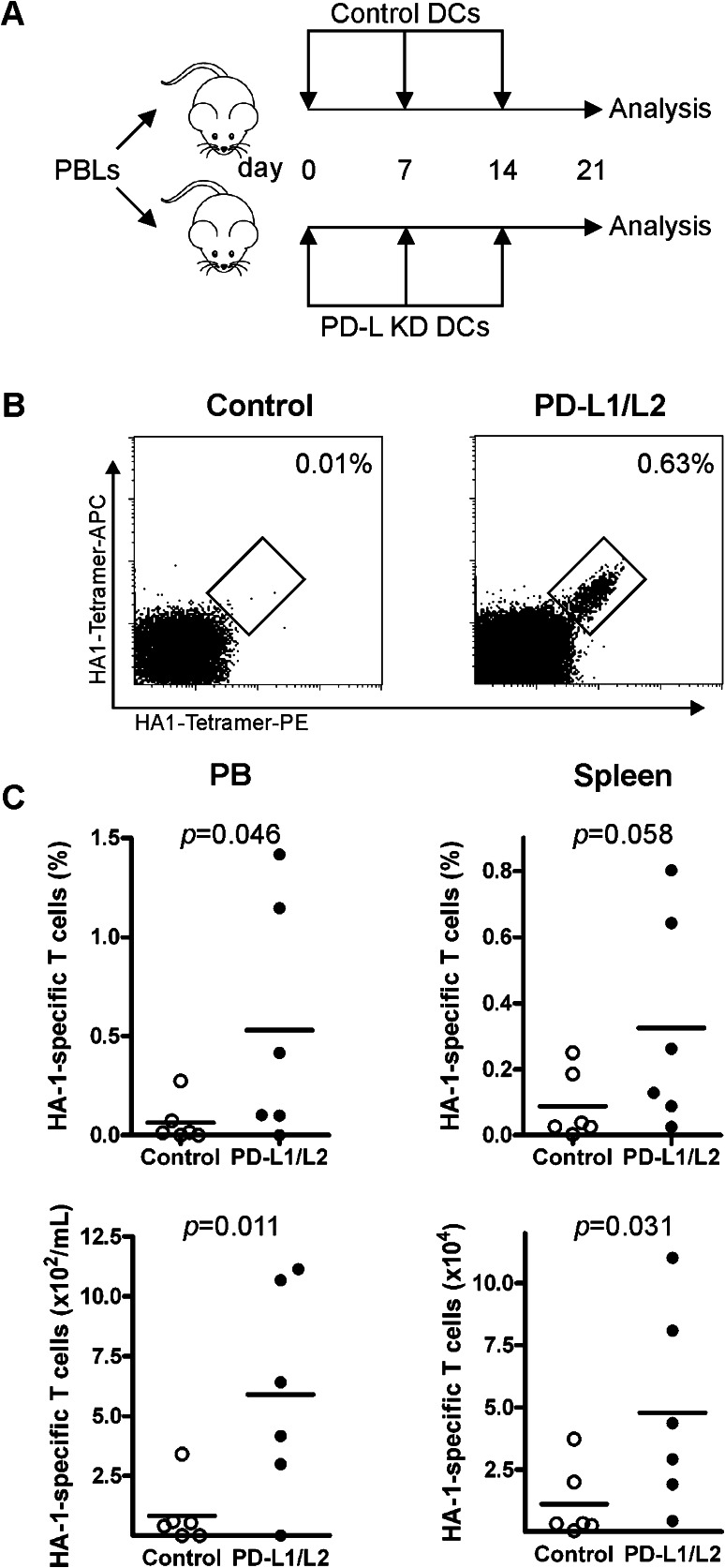
PD-L silenced DCs enhanced MiHA-specific CD8^+^ T cell expansion in vivo. **a** HA1-primed PBLs containing 2900 HA1-specific T cells were injected in NSG mice and vaccinated three times with control or PD-L silenced (relative expression: PD-L1 13.1 %, PD-L2 10.5 %) DCs at weekly intervals. Mice were killed at day 21, and peripheral blood and spleen were analyzed by flow cytometry for human HA-1-specific CD8^+^ T cells. **b** Representative FACS plot of HA-1-specific CD8^+^ T cells in spleen after control or PD-L silenced DC vaccinations. The *numbers* in the FACS plots represent the percentage of HA-1-specific CD8^+^ T cells within the CD3^+^CD8^+^ T cell population. **c** Percentages within CD8^+^ T cells, and absolute levels of HA-1-specific CD8^+^ T cells in peripheral blood and spleen. *Each dot* represents a single mouse, *n* = 6 mice per group. Statistical analysis was performed using a one-tailed Student’s *t* test

Next, we addressed the potency of PD-L silenced DCs to superiorly boost the expansion of adoptively transferred MiHA-specific CD8^+^ T cells in vivo. Therefore, MiHA-primed PBLs were injected in NSG mice followed by weekly vaccinations with either control or PD-L silenced DCs (Fig. 4a). After 3 weeks, mice were killed. We observed similar engraftment levels of human CD45^+^ and CD8^+^ T cells in mice vaccinated with control or PD-L silenced DCs (Supplementary Figure 2). During the experiment, no signs of GVHD were observed, as weights of the mice were stable and no differences in fur appearance, activity or statue were noticed between control and PD-L silenced DCs (data not shown). Interestingly, while mice vaccinated with control DCs showed only low levels of MiHA-specific CD8^+^ T cells (0.06 ± 0.11 %), PD-L silenced DCs expanded the MiHA-specific CD8^+^ T cell numbers up to 1.5 % in PB (0.53 ± 0.60 %, *p* = 0.046, Fig. 4b–c). Also, in spleen this increase in MiHA-specific CD8^+^ T cells by PD-L silenced DCs was observed, though did not reach significance (0.09 ± 0.10 % in case of control DCs vs. 0.32 ± 0.32 % in case of PD-L silenced DCs, *p* = 0.058). Nevertheless, absolute numbers of MiHA-specific CD8^+^ T cells were significantly augmented in mice vaccinated with PD-L silenced DCs, in both spleen and PB (83 ± 129 vs. 590 ± 440/ml in PB, *p* = 0.011; and 1.1 × 10^4^ ± 1.5 × 10^4^ vs. 4.8 × 10^4^ ± 4.0 × 10^4^ in spleen, *p* = 0.031, Fig. 4b–c). This resulted in a 4- versus 17-fold expansion of the MiHA-specific CD8^+^ T cells in control DC- versus PD-L silenced DC-treated mice, respectively. This demonstrated that PD-L silenced DCs exhibit enhanced stimulatory capacity to boost the expansion of adoptively transferred MiHA-specific CD8^+^ T cells in vivo.

## Discussion

Despite the curative potential of allo-SCT, many patients relapse. In previous studies, we have demonstrated that the frequencies of productive T cell responses in these patients are inadequate. This emphasizes the need for additive therapy to improve GVT immunity and consequently overall survival of patients suffering from hematological malignancies. To reduce the risk of GVHD, these additive therapies should exploit MiHA-specific CD8^+^ T cells recognizing antigens expressed solely by the hematopoietic system. By this, selective GVT immunity could be boosted without promoting GVHD.

Currently, additive T cell therapy can be given post-allo-SCT as DLI. Yet, these non-selected donor lymphocytes contain only few tumor-reactive T cells, while a substantial percentage of T cells could contribute to the life-threatening complication GVHD. Ex vivo priming of donor lymphocytes would enrich the DLI for GVT-specific MiHA-specific CD8^+^ T cells. Although priming of MiHA-specific CD8^+^ T cells can occur during pregnancy [25], the MiHA-specific CD8^+^ T cell responses in our assays most likely emerged from the naive T cell repertoire, as confirmed by the absence of MiHA-specific CD8^+^ T cell responses in case effector memory T cells, were used as starting material. Notably, the extent of the ex vivo primed MiHA-specific CD8^+^ T cell responses varied between donors, probably due to variation in precursor frequencies between donors and different MiHAs [26, 27]. In this manuscript, we are the first to demonstrate that the use of PD-L silenced DCs in these ex vivo cultures greatly enhanced the priming and expansion of MiHA-specific CD8^+^ T cell responses. Notably, we recently demonstrated that ex vivo priming of the MiHA-specific CD8^+^ T cells for adoptive T cell transfer could be further improved by retaining these cells in an early memory state using a selective Akt-inhibitor during ex vivo culture [28].

DC vaccination is a strong tool to boost adoptively transferred MiHA-specific CD8^+^ T cells. To improve the activation and expansion of these tumor-reactive T cells by DC-based stimulation, one can interfere with the co-inhibitory PD-1/PD-L pathway. Though recent papers showed clinical effects of PD-1 blockade in solid tumors and after autologous stem cell transplantation without causing severe side effects [29–31], speculations have been made on the risk of these blocking antibodies on GVHD. Addition of PD-1/PD-L1 blocking antibodies to the activated immune system early after allo-SCT could further release the brake on all activated T cells, resulting in the induction and aggravation of GVHD. Therefore, we believe that PD-L silenced DCs are a safer way to interfere with the PD-1/PD-L pathway in the allo-SCT setting, and thereby potently augment the activation and expansion of MiHA-specific CD8^+^ T cells. The use of DC vaccinations to boost tumor-reactive T cells is a safe and feasible application as shown in several DC vaccination trials in cancer patients [32]. Applying adoptive transfer of hematopoietic-restricted MiHA-specific CD8^+^ T cells in combination with potent PD-L silenced DC vaccines could augment GVT immunity and thereby prevent relapse and improve survival.

We previously showed the potential of PD-L silenced DCs in the expansion of MiHA-specific CD8^+^ effector memory T cells in vitro [11, 12]. In this paper, we are the first to demonstrate that PD-L silenced DCs are also superior in boosting antigen-specific CD8^+^ T cells in vivo. Moreover, in addition to our superior expansion, Pen et al. [33] recently showed improved cytokine production of T cells upon interference with the PD-1/PD-L1 pathway. This was also shown by Lesterhuis et al. [34], who demonstrated that upon down-regulation of PD-L1 and PD-L2 expression on DCs by platinum-based chemotherapeutics, antigen-specific T cells show enhanced proliferation and Th1 cytokine secretion. In addition, DNA vaccination of soluble PD-1, which interfered with the natural PD-1/PD-L1 interaction, resulted in an increased functionality and antitumor effect in mice [35]. More promisingly, Ge et al. [36] showed that the use of a PD-L1 blocking antibody during ex vivo priming of antigen-specific T cells and subsequent DC vaccination resulted in a better cytokine production and expansion of these T cells in vitro, and an improved anti-tumor effect in vivo. All together, these observations illustrate the potency of PD-L silenced DCs with adoptive transfer of MiHA-specific CD8^+^ T cells, which should be further explored for improving an antitumor effect in a relevant tumor therapy model. However, first a robust and appropriate isolation method to purify ex vivo primed MiHA-specific CD8^+^ T cells before adoptive transfer into tumor-bearing mice should be set up. This could be performed by the Streptamer technique obtaining viable MiHA-specific CD8^+^ T cells for adoptive immunotherapy [37].

Our current data illustrate a novel additive value of our approach in relation to previous reports. This therapeutic strategy would especially be attractive for clinical exploration in patients who lack productive tumor-reactive T cell responses. The combination of adoptive transfer of ex vivo primed MiHA-specific CD8^+^ T cells with PD-L silenced DCs vaccines targeting hematopoietic-restricted MiHAs would greatly contribute to better GVT immunity without inducing detrimental GVHD. This preemptive therapy could prevent relapse and thereby improve survival in allo-SCT patients. Furthermore, our strategy could be broadly exploited toward tumor-antigen or virus-specific CD8^+^ T cells in cancer and viral infections.

### Electronic supplementary material

Below is the link to the electronic supplementary material.

Supplementary material 1 (PDF 304 kb)

## Abbreviations

Allo-SCT: Allogeneic stem cell transplantation
ANOVA: Analysis of variance
BSA: Bovine serum albumin
CMV: Cytomegalovirus
DC: Dendritic cell
DLI: Donor lymphocyte infusion
GM-CSF: Granulocyte macrophage colony-stimulating factor
GVHD: Graft-versus-host disease
GVT: Graft-versus-tumor
HLA: Human leukocyte antigen
HS: Human serum
IL: Interleukin
MiHA: Minor histocompatibility antigen
NSG: NOD/SCID/IL2Rγ^null^
PB: Peripheral blood
PBLs: Peripheral blood lymphocytes
PBMCs: Peripheral blood mononuclear cells
PD-1: Program death-1
PD-L: Program death ligand
PFA: Paraformaldehyde
PGE2: Prostaglandin E2
RFS: Relapse-free survival
Tem: Effector memory T
Tn: Naive T
TNF: Tumor necrosis factor
