# Supplementary material for: siRNA silencing of PD-1 ligands on dendritic cell vaccines boosts the expansion of minor histocompatibility antigen-specific CD8+ T cells in NOD/SCID/IL2Rg(null) mice
Source: Cancer Immunol Immunother. 2015 Feb 28;64(5):645–54. doi: 10.1007/s00262-015-1668-6 (PMC4412509; doi:10.1007/s00262-015-1668-6)
Supplement: Supplementary file 1 — Supplementary material 1 (PDF 304 kb) [file 262_2015_1668_MOESM1_ESM.pdf]

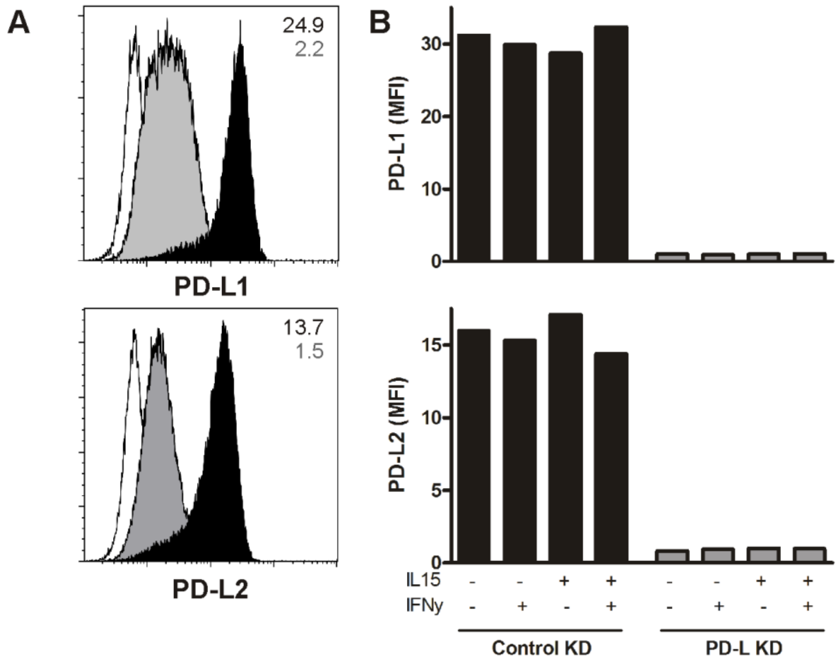

Supplementary Figure 1. Stable PD-L silencing on dendritic cells. (**A**) Representative FACS plots of PD-L1 and PD-L2 expression on control (black) or PD-L silenced (grey) DCs at the end of their maturation culture. White histogram represent isotype control staining. The numbers in the FACS plots represent the mean fluorescence intensity (MFI). (**B**) These DCs were subsequently cultured in maturation medium with or without IFNγ (100 U/ml) and/or IL15 (5 ng/ml) for 2 days. MFI of PD-L1 and PD-L2 expression are corrected for isotype control.

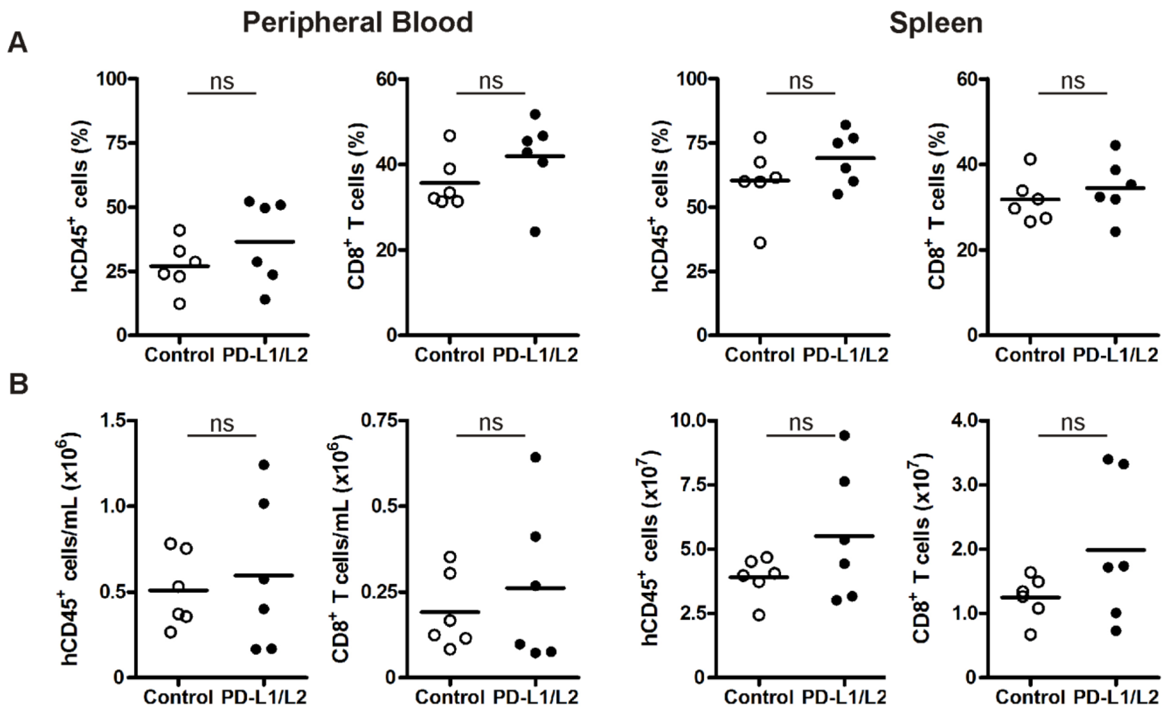

Supplementary Figure 2. Engraftment levels of human cells after adoptive T cell transfer and vaccination with control or PD-L silenced DCs in NSG mice. PBLs were primed using HA-1-loaded DCs for 7 days, after which NSG mice were injected with 10 x 10^6 cells. Then they were vaccinated three times with control or PD-L silenced (relative expression: PD-L1 13%, PD-L2 11%) DCs at weekly intervals. Mice were sacrificed at day 21. (**A**) Percentage and (**B**) absolute numbers of human CD45 (% of total human and mouse $CD45^+$ cells) and $CD8^+$ T cells (% within human $CD45^+$ cells). Each dot represents a single mouse, *n*=6 mice per group. Statistical analysis was performed using an one-tailed student t-test.
